# Olanzapine enhances the response of PD-(L)1 inhibitor immunotherapy: A retrospective efficacy analysis in advanced malignancies

**DOI:** 10.1016/j.isci.2026.115568

**Published:** 2026-04-01

**Authors:** Yan-ling Yi, Meng-xue Mei, Hai-hui Wang, Jiang-zhe Ye, Zhen-jie Huang, Sha Zhao, An-wen Liu, Long Huang

**Affiliations:** 1 Department of Lung Cancer Center, The Second Affiliated Hospital of Nanchang University, 1Minde Road, Nanchang, Jiangxi, China; 2 Department of Oncology, The Second Affiliated Hospital of Nanchang University, 1Minde Road, Nanchang, Jiangxi, China

**Keywords:** oncology, therapeutics

## Abstract

This retrospective cohort study explores olanzapine’s immunomodulatory role and clinical impact in patients with advanced cancer on PD-(L)1 inhibitors, given that chronic stress may impair immune checkpoint inhibitor (ICI) efficacy. Among 1933 patients with advanced cancer receiving PD-(L)1 inhibitors + chemotherapy (2018–2022), 99 received concurrent olanzapine for chemotherapy-induced nausea. After excluding 58 cases, 41 olanzapine-treated patients were propensity score-matched (PSM) for age, gender, and cancer type. Survival outcomes were analyzed via Kaplan-Meier curves and log rank tests. The olanzapine cohort showed higher objective response rate (ORR) (39.02% vs. 26.83%), comparable median PFS (9 vs. 6 months, *p* = 0.057), and significantly longer median OS (28 vs. 9 months, *p* = 0.019). Multivariate analysis confirmed olanzapine correlated with prolonged OS (HR = 0.510, 95% CI:0.282–0.917, *p* = 0.026). Olanzapine may enhance survival benefits in patients with advanced cancer undergoing PD-(L)1 inhibitor therapy.

## Highlights

• Olanzapine possesses neuroimmunomodulatory properties
• Olanzapine improves the objective response rate (ORR) of PD-1 monoclonal antibody therapy
• Olanzapine enhances survival benefits in patients with cancer undergoing PD-(L)1 inhibitor therapy

## Graphical abstract

**Figure:**
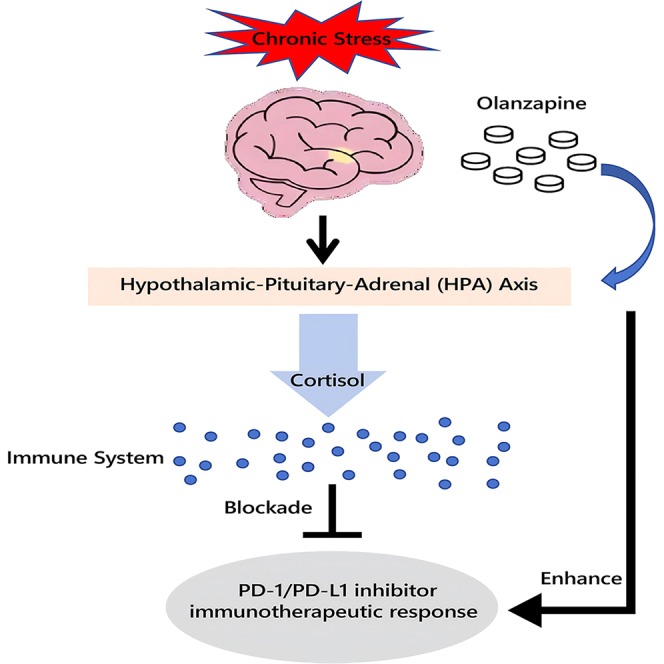

## Introduction

Immune checkpoint inhibitors (ICIs), particularly PD-(L)1 monoclonal antibodies, have emerged as the fourth cornerstone of cancer therapy alongside surgery, chemotherapy, and radiotherapy, significantly improving survival outcomes in advanced malignancies.^1^ However, substantial heterogeneity in treatment response persists, with efficacy modulated by complex host-intrinsic and extrinsic factors. ICIs-mediated immunotherapy has only 20%–30% of patients benefit from it.^2^

Emerging evidence indicates that chronic psychological stress—a critical microenvironmental variable—may impede antitumor immunity via neuroendocrine immune axis dysregulation, thereby diminishing clinical benefits from ICIs.^3^^,^^4^^,^^5^ Previous studies have indicated that the incidence of depression among patients with cancer is increasing annually and is over five times higher than that of the general population.^6^ Concurrently, depression significantly reduces the quality of life and increases mortality in patients with malignant tumors.^7^ The STRESS-LUNG-1 prospective study demonstrated that in patients with advanced non-small cell lung cancer (NSCLC) treated with ICIs, higher levels of emotional distress (ED) are associated with a worse therapeutic response to these inhibitors.^3^ Furthermore, numerous studies have shown that combining ICIs with other cancer therapies enhances clinical responses and exerts synergistic effects in patients with various cancer types.^8^

Emerging evidence from studies suggests that antidepressants may exert beneficial effects beyond their established role in alleviating anxiety and depression in patients with cancer. These agents have been shown to modulate multiple signaling pathways and influence tumor microenvironment (TME) dynamics, potentially contributing to direct anti-tumor activity and mitigation of chemotherapy-induced side effects.^9^ Notably, preclinical research has demonstrated that fluoxetine, through antagonism of serotonin signaling, can downregulate tumor PD-L1 expression in murine models. This immunomodulatory effect enhances the efficacy of ICIs, resulting in significantly suppressed tumor progression when used in combination with immunotherapy.^10^ As research advances, growing experimental and translational data support the synergistic potential of combining antidepressants with conventional anticancer treatments. Such combinations appear to enhance antitumor responses by promoting immune system activation, alleviating cancer-related symptoms, improving patients’ quality of life, and potentially extending survival, highlighting a novel, multidimensional therapeutic role for these drugs in oncology. Despite these promising insights, robust clinical evidence evaluating the impact of antidepressant use on immunotherapy outcomes in human cancer populations remains sparse. Well-designed prospective trials are therefore needed to clarify whether these observed preclinical benefits translate into tangible clinical improvements, and to identify optimal patient subgroups, timing, and specific antidepressant classes for integration into cancer immunotherapeutic strategies.

Given the widespread use of antipsychotics (for example, olanzapine) for managing cancer-related symptoms such as chemotherapy-induced nausea, insomnia, and anxiety, their potential immunomodulatory properties warrant rigorous investigation. To address this gap, we conducted a retrospective cohort analysis evaluating the association between adjunctive olanzapine use and clinical outcomes in patients with advanced cancer receiving PD-(L)1 inhibitor therapy.

## Results

### Patient characteristics

**Figure 1:**
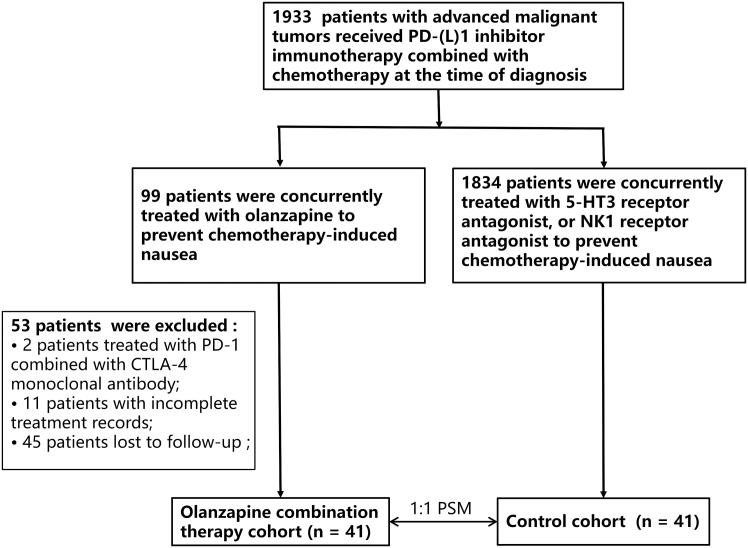
Flow diagram of the study

Forty-one patients with advanced cancer administered olanzapine plus PD-(L)1 inhibitors were enrolled. A propensity score-matched (PSM)-matched control cohort (*n* = 41) received first-line PD-(L)1 inhibitors without olanzapine (Figure 1). It can be seen that the baseline factors were not significantly different between the olanzapine combination therapy cohort and the control cohort (all *p* > 0.05) (Table 1). All patients included in this study received prophylactic olanzapine to prevent chemotherapy-induced gastrointestinal reactions. The administration schedule was as follows: oral administration of 5 mg once daily on days 1–5 of each chemotherapy cycle. The median treatment time for olanzapine was 14 days (range 5–67 days) in the olanzapine combination therapy cohort. The median age was 63 years (range 34–75 years).

**Table 1:** Clinical characteristics of patients who received olanzapine combination PD-(L)1 inhibitor therapy cohort and control cohort after PSM

Characteristic	Total (*n* = 82)	Olanzapine combination therapy cohort (*n* = 41)	Control cohort (*n* = 41)	*p* value
Sex, No(%)	–	–	–	0.800
Male	61(74.4)	31(75.6)	30(73.2)	–
Female	21(25.6)	10(24.4)	11(26.8)	–
Age, No(%)	–	–	–	0.969
<50	11(13.4)	6(14.6)	5(12.2)	–
50-70	45(54.9)	22(53.7)	23(56.1)	–
>70	26(31.7)	13(31.7)	13(31.7)	–
BMI, No(%)	–	–	–	0.596
<18	5(6.1)	3(7.3)	2(4.9)	–
18-24	61(74.4)	30(73.2)	31(75.6)	–
>24	16(19.5)	8(19.5)	8(19.5)	–
Smoker, No(%)	–	–	–	0.800
Yes	21(25.6))	10(24.4)	11(26.8)	–
No	61(74.4)	31(75.6)	30(73.2)	–
Alcoholic, No(%)	–	–	–	0.577
Yes	16(19.5)	7(17.1)	9(22.0)	–
No	66(80.5)	34(82.9)	32(78.0)	–
PS, No(%)	–	–	–	1.000
0	3(3.7)	0(0.0)	3(7.3)	–
1∼2	79(96.3)	41(100.0)	38(92.7)	–
3∼4	0(0.0)	0(0.0)	0(0.0)	–
Type of tumor, No(%)	–	–	–	0.965
Lung cancer	35(42.7)	18(43.9)	17(41.5)	–
Gastrointestinal cancer	16(19.5)	8(19.5)	8(19.5)	–
Head and neck cancers	14(17.1)	7(17.1)	7(17.1)	–
Esophageal cancer	6(7.3)	3(7.3)	3(7.3)	–
Cervical cancer	4(4.9)	2(4.9)	2(4.9)	–
Urothelial carcinoma	4(4.9)	2(4.9)	2(4.9)	–
Liver cancer	3(3.7)	1(2.4)	2(4.9)	–
Chemotherapy cycles, No(%)	–	–	–	0.537
≥4	70(85.4)	36(87.8)	34(82.9)	–
<4	12(14.6)	5(12.2)	7(17.1)	–

### Survival outcomes

**Figure 2:**
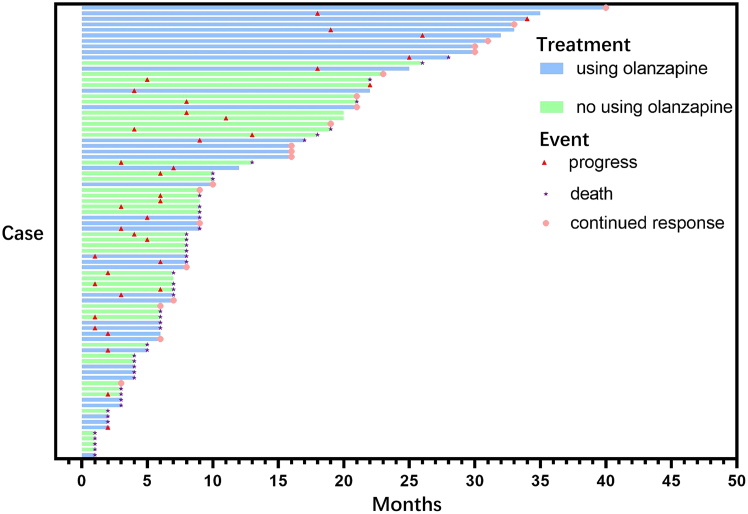
Swimming plot illustrates the treatment history of patients

**Figure 3:**
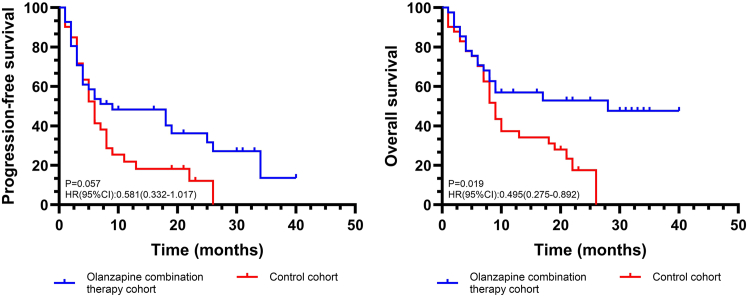
Kaplan-Meier curve and subgroup analysis of PFS and OS *p* values were calculated using a two-sided log rank test. The HR and the corresponding 95% CI were calculated using a Cox proportional-hazards.

The objective response rate (ORR) in the olanzapine combination therapy cohort and control cohort was 39.02% (16/41) vs. 26.83% (11/41), respectively. The disease control rate (DCR) in the olanzapine combination therapy cohort and control cohort was 73.17% (30/41) vs. 63.41% (26/41), respectively. As of the follow-up endpoint, 18 deaths were recorded in the olanzapine combination therapy group versus 30 in the control group. Regarding continuous disease remission, 14 patients in the combination therapy group achieved this outcome, compared with only 5 in the control group (Figure 2). Kaplan-Meier survival curve analysis showed patients with olanzapine combination therapy have significantly longer median OS than the control cohort (mOS: 28 vs. 9 months; HR: 0.495 (95%CI: 0.275–0.892); *p* = 0.019). The survival analysis showed that patients with olanzapine combination therapy have longer median PFS than the control cohort (mPFS: 9 vs. 6 months; HR: 0.581 (95%CI: 0.332–1.017)), but it is not statistically significant (Figure 3). Multivariate analysis confirmed that olanzapine use was significantly associated with prolonged OS in patients receiving PD-(L)1 inhibitors immunotherapy (HR = 0.510, 95% CI: 0.282–0.917, *p* = 0.026) (Data S1).

## Discussion

For many patients with advanced malignancies, factors including treatment confidence, toxic side effects, and economic pressures contribute to chronic psychological stress, often manifesting as depression and anxiety. Extensive preclinical evidence indicates that psychological states significantly influence cancer progression and the efficacy of immunotherapy. The most widely accepted mechanism posits that ED impairs immune surveillance and anti-tumor immune responses through neuroendocrine pathways mediated by β-adrenergic/glucocorticoid signaling.^11^ Chronic stress induces persistent dysregulation of the neuroendocrine system, leading to the sustained release of stress hormones such as glucocorticoids, epinephrine, and norepinephrine.^12^ These hormones activate corresponding signaling pathways, alter the TME, and suppress the activation, infiltration, and cytotoxic function of CD8^+^ T cells.^13^ Consequently, this cascade undermines the response to PD-1 blockade therapy,^14^ compromises immune surveillance and inhibition, and ultimately promotes tumor initiation, progression, metastasis, and multidrug resistance-a key factor contributing to immunotherapy failure.

A recent post hoc analysis of the Phase II PRADO trial investigated the association between pretreatment ED and the efficacy of neoadjuvant immunotherapy in patients with melanoma. The analysis revealed that compared with the no-ED group, the baseline ED group showed a lower major pathological response rate (46% vs. 65%) and reduced 2-year relapse-free survival (74% vs. 91%).^15^ Furthermore, a study by Fang Wu’s team on patients with advanced NSCLC also indicated a link between ED and diminished response to ICIs. The study reported significantly shorter progression-free survival in the ED group and a higher risk of overall survival events, supporting that psychological stress is associated with poorer efficacy of ICIs in advanced NSCLC.

These studies and findings suggest a potential link between psychological status and the efficacy of immunotherapy. In recent years, a growing body of preclinical research has explored the effects of antidepressant drugs on cancer. One study demonstrated that sertraline could sensitize human lung cancer cells to tumor necrosis factor-related apoptosis-inducing ligand (TRAIL, a potential target in cancer therapy) and may also serve as a treatment option for patients with cancer with depression.^16^ Another study reported that fluoxetine, by antagonizing serotonin in mice, altered the TME, leading to decreased PD-L1 expression in tumors, slowed tumor growth, and, when combined with immunotherapy, induced prolonged arrest of cancer progression.^10^ However, clinical evidence regarding the association between antidepressant use and immunotherapy outcomes remains limited.

In this study, we observed that patients receiving olanzapine in combination with ICIs exhibited a significantly longer median OS compared to those in the control cohort. Additionally, the combination group demonstrated a higher ORR, suggesting that olanzapine may enhance the therapeutic efficacy of ICIs in patients with advanced tumors. These findings further support the hypothesis that targeting the ED could modulate the tumor immune microenvironment and potentially improve ICI outcomes, implicating patients’ psychological status as a potential predictive biomarker for immunotherapy response. Olanzapine may enhance the efficacy of immunotherapy through dual pathways: it reshapes the balance of Th1/Th2 subsets, promotes the secretion of Th1-type cytokines such as IFN-γ, and enhances the tumor-killing activity of CTLs; meanwhile, it inhibits the immunosuppressive function of Treg cells, releasing their “brake” effect on effector T cells. Olanzapine reduces the levels of pro-inflammatory factors such as IL-6 and TNF-α in the TME to alleviate immunosuppression; it also upregulates the concentrations of pro-immunostimulatory cytokines such as IL-2 and IL-12, enhancing the antigen-presenting ability of DC cells. These mechanisms collectively reverse the tumor immunosuppressive microenvironment and synergize with ICIs. However, there are studies in cancer showing the direct effects of olanzapine itself.^17^^,^^18^ Furthermore, olanzapine may benefit patients with cancer not only as an immunomodulator, but also by reducing chemoresistance through down-regulation of survivin.^19^

### Limitations of the study

Despite these promising results, several limitations warrant consideration. First, the retrospective and non-randomized design of the study inherently carries a risk of selection bias. Second, our cohort encompassed multiple tumor types rather than being restricted to a single cancer lineage. While this broad inclusion enhances generalizability, it also introduces heterogeneity due to variations in tumor biology and inherent responsiveness to immunotherapy across different malignancies. Therefore, future large-scale, prospective, and ideally randomized studies are needed to validate these findings and elucidate the underlying mechanisms by which psychotropic interventions such as olanzapine may synergize with ICIs.

## Resource availability

### Lead contact

Further information and requests for resources and reagents should be directed to and will be fulfilled by the lead contact, Long Huang (ndefy13211@ncu.edu.cn).

### Materials availability

This study did not generate new unique reagents.

### Data and code availability

• Data: This paper does not report a new dataset.
• Code: This paper does not report original code.
• All other items: any additional information required to reanalyze the data reported in this paper is available from the lead contact upon request.

## Acknowledgments

The authors are grateful to all the patients and their families involved in the study. This work was supported by the 10.13039/501100004479Jiangxi Provincial Natural Science Foundation, Grant/Award Numbers: 20242BAB25550; Wu Jieping Foundation of China [grant number: 320.6750.2025-06-233].

## Author contributions

Conceptualization, L.H. and A.W.L.; methodology, Y.L.Y. and M.X.M.; formal analysis, Z.J.H. and S.Z.; investigation, H.H.W. and J.Z.Y.; resources, L.H.; writing – original draft, Y.L.Y. and M.X.M.; visualization, L.H.; supervision, A.W.L.; project administration, L.H.; funding acquisition, L.H.

## Declaration of interests

The authors declare no competing interests.

## STAR★Methods

### Key resources table

**Table:** 

REAGENT or RESOURCE	SOURCE	IDENTIFIER
**Software and algorithms**		

SPSS Statistics software (version 26.0).	This paper	https://www.ibm.com/support/pages/downloading-ibm-spss-statistics-26-transition-extended-support-30-sep-2025
GraphPad Prism	GraphPad Inc.	https://www.graphpad.com

### Experimental model and study participant details

All patients enrolled in this study were Asian (Han Chinese), including 67 males and 21 females. Patient details can be found in Data S2. We analyzed the effect of sex on the study outcomes, and the results showed no significant difference between the two groups. This study was performed in line with the principles of the Declaration of Helsinki. The study was reviewed and approved by the Ethics Committee of the Second Affiliated Hospital of Nanchang University. The ethical approval number is I-2025-132. During hospitalization, each patient signed a hospital-universal broad informed consent form, agreeing that their excess biological samples from clinical tests and clinical diagnosis and treatment data could be used for scientific research.

### Method details

From January 2018 to December 2022, a total of 1933 treatment-naive patients with advanced malignant tumors received PD-(L)1 inhibitor immunotherapy combined with chemotherapy at the Second Affiliated Hospital of Nanchang University, among whom 99 were concurrently treated with olanzapine to prevent chemotherapy-induced nausea. A total of 58 cases were excluded, including 2 patients treated with PD-1 monoclonal antibody combined with CTLA-4 monoclonal antibody, 11 patients with incomplete treatment records, and 45 patients lost to follow-up (Figure 1). To analyze survival outcomes in patients receiving PD-(L)1 inhibitor plus olanzapine, we adjusted for age, gender, smoking history, BMI, PS (Performance Status score), and cancer type using propensity score matching (PSM) .

The endpoints in this study were overall survival and progression-free survival. The median follow-up time in this study was 13 months (range 1-41 months). The date of last follow-up was May 10, 2025. Progression-free survival (PFS) was defined as the time from the date of PD-(L)1 inhibitors therapy initiation to the date of disease progression. The disease progression was based on radiological evaluation and/or clinical progression using the Response Evaluation Criteria in Solid Tumors criteria (RECIST) (version 1.1). Overall survival (OS) was defined as the time from the date of PD-(L)1 inhibitors therapy initiation after diagnosis as advanced malignant tumors to the date of death for any reason.

### Quantification and statistical analysis

Descriptive statistics were used to summarize baseline patient characteristics. To eliminate the effect of confounding covariates on survival analysis, PSM was performed using one-to-one nearest neighbor matching. The Kaplan–Meier survival curves and log-rank test were used to analyze OS and PFS. Cox proportional hazards regression model was utilized to calculate the hazard ratios (HRs) and their 95% confidence intervals (CIs). All variables were taken in the multivariate logistic regression analysis model. All p-values indicated were calculated with a Student’s t-Test. All t-Tests calculated were two-tailed and unpaired with equal variance unless otherwise specified. P value< 0.05 was considered statistically significant. All statistical analyses were performed using SPSS Statistics software (version 26.0). GraphPad Prism (version 10.4.1) was used to produce graphical representations of data.
