# Supplementary material for: Olanzapine enhances the response of PD-(L)1 inhibitor immunotherapy: A retrospective efficacy analysis in advanced malignancies
Source: iScience. 2026 Apr 1;29(5):115568. doi: 10.1016/j.isci.2026.115568 (PMC13099940; doi:10.1016/j.isci.2026.115568)
Supplement: Document S1. Data S1 and S2 [file mmc1.pdf]

iScience, Volume *29*

Supplemental information

Olanzapine enhances the response of PD-(L)1 inhibitor immunotherapy: A retrospective efficacy analysis in advanced malignancies

Yan-ling Yi, Meng-xue Mei, Hai-hui Wang, Jiang-zhe Ye, Zhen-jie Huang, Sha Zhao, An-wen Liu, and Long Huang

Data S1: Multivariate COX Regression Analysis of OS Risk Factors.

variables	HR	95% CI	P
Sex	1.446	0.773-2.691	0.257
Age	1.023	0.962 -1.254	0.093
BMI	0.944	0.833-1.065	0.302
Smoke	1.002	0.532-1.903	0.989
Alcoholic	1.441	0.684-3.092	0343
PS	0.675	0.215-2.167	0.498
Type of tumor	1.052	0.486-2.321	0.896
Chemotherapy cycles	1.124	0.832-2.514	0.451
Olanzapine use	0.510	0.282-0.917	0.026

Data S2: Cancer type and medication regimen of the patient.

NO.	Group	Cancer type	PD-1/PDL-1	Chemotherapy regimen
1	1	Non small cell lung cancer	Tislelizumab	Paclitaxel + Carboplatin
2	1	Head and neck cancer	Camrelizumab	Gemcitabine + Cisplatin
3	1	Head and neck cancer	Pembrolizumab	Paclitaxel
4	1	Head and neck cancer	Tislelizumab	Paclitaxel + Carboplatin
5	1	Esophageal cancer	Pembrolizumab	Paclitaxel + Carboplatin
6	1	Non small cell lung cancer	Toripalimab	Paclitaxel + Carboplatin
7	1	Cervical cancer	Durvalumab	Paclitaxel + Cisplatin
8	1	Non small cell lung cancer	Tislelizumab	Paclitaxel + Carboplatin
9	1	Gastric cancer	Nivolumab	Paclitaxel + Cisplatin
10	1	Gastric cancer	Toripalimab	Paclitaxel + Cisplatin
11	1	Non small cell lung cancer	Tislelizumab	Paclitaxel + Carboplatin
12	1	Non small cell lung cancer	Tislelizumab	Paclitaxel + Carboplatin
13	1	Esophageal cancer	Pembrolizumab	Paclitaxel
14	1	Esophageal cancer	Nivolumab	Paclitaxel + Carboplatin
15	1	Small cell lung cancer	Durvalumab	Etoposide + Cisplatin
16	1	Non small cell lung cancer	Camrelizumab	Paclitaxel + Carboplatin
17	1	Gastric cancer	Camrelizumab	Paclitaxel + Carboplatin
18	1	Non small cell lung cancer	Tislelizumab	Pemetrexed + Carboplatin
19	1	Gastric cancer	Nivolumab	Paclitaxel

20	1	Non small cell lung cancer	Tislelizumab	Pemetrexed + Carboplatin
21	1	Cervical cancer	Tislelizumab	Paclitaxel + Cisplatin
22	1	Urothelial carcinoma	Tislelizumab	Gemcitabine + Cisplatin
23	1	Non small cell lung cancer	Tislelizumab	Pemetrexed
24	1	Non small cell lung cancer	Toripalimab	Paclitaxel + Carboplatin
25	1	Small cell lung cancer	Durvalumab	Etoposide + Cisplatin
26	1	Non small cell lung cancer	Tislelizumab	Pemetrexed + Carboplatin
27	1	Urothelial carcinoma	Tislelizumab	Gemcitabine + Cisplatin
28	1	Liver cancer	Pembrolizumab	Oxaliplatin + Fluorouracil
29	1	Head and neck cancer	Toripalimab	Paclitaxel + Cisplatin
30	1	Non small cell lung cancer	Tislelizumab	Pemetrexed
31	1	Non small cell lung cancer	Toripalimab	Pemetrexed + Carboplatin
32	1	Gastric cancer	Toripalimab	Paclitaxel + Carboplatin
33	1	Colon cancer	Nivolumab	Oxaliplatin + Fluorouracil
34	1	Non small cell lung cancer	Tislelizumab	Pemetrexed + Carboplatin
35	1	Non small cell lung cancer	Camrelizumab	Pemetrexed + Carboplatin
36	1	Non small cell lung cancer	Tislelizumab	Pemetrexed + Carboplatin
37	1	Head and neck cancer	Pembrolizumab	Gemcitabine + Cisplatin
38	1	Head and neck cancer	Toripalimab	Paclitaxel + Cisplatin
39	1	Gastric cancer	Nivolumab	Paclitaxel + Carboplatin
40	1	Head and neck cancer	Pembrolizumab	Paclitaxel + Cisplatin
41	1	Gastric cancer	Tislelizumab	Paclitaxel + Carboplatin
42	2	Head and neck cancer	Tislelizumab	Paclitaxel + Cisplatin
43	2	Non small cell lung cancer	Tislelizumab	Pemetrexed + Carboplatin
44	2	Non small cell lung cancer	Tislelizumab	Paclitaxel + Cisplatin
45	2	Head and neck cancer	Pembrolizumab	Paclitaxel + Cisplatin
46	2	Small cell lung cancer	Durvalumab	Etoposide + Cisplatin
47	2	Small cell lung cancer	Durvalumab	Etoposide + Cisplatin
48	2	Cervical cancer	Tislelizumab	Paclitaxel + Cisplatin
49	2	Non small cell lung cancer	Pembrolizumab	Paclitaxel
50	2	Urothelial carcinoma	Tislelizumab	Gemcitabine + Cisplatin
51	2	Non small cell lung cancer	Toripalimab	Pemetrexed + Carboplatin
52	2	Colon cancer	Camrelizumab	Oxaliplatin + Fluorouracil
53	2	Esophageal cancer	Camrelizumab	Paclitaxel
54	2	Non small cell lung cancer	Tislelizumab	Paclitaxel
55	2	Non small cell lung cancer	Pembrolizumab	Pemetrexed + Carboplatin

56	2	Non small cell lung cancer	Tislelizumab	Paclitaxel + Carboplatin
57	2	Gastric cancer	Nivolumab	Paclitaxel + Carboplatin
58	2	Head and neck cancer	Toripalimab	Paclitaxel + Cisplatin
59	2	Head and neck cancer	Pembrolizumab	Paclitaxel + Carboplatin
60	2	Non small cell lung cancer	Tislelizumab	Paclitaxel + Carboplatin
61	2	Non small cell lung cancer	Toripalimab	Paclitaxel + Carboplatin
62	2	Urothelial carcinoma	Tislelizumab	Gemcitabine + Cisplatin
63	2	Gastric cancer	Nivolumab	Paclitaxel + Carboplatin
64	2	Gastric cancer	Nivolumab	Paclitaxel + Carboplatin
65	2	Non small cell lung cancer	Toripalimab	Paclitaxel + Carboplatin
66	2	Head and neck cancer	Durvalumab	Paclitaxel + Capecitabine
67	2	Non small cell lung cancer	Toripalimab	Gemcitabine + Cisplatin
68	2	Non small cell lung cancer	Toripalimab	Pemetrexed + Carboplatin
69	2	Gastric cancer	Nivolumab	Paclitaxel + Carboplatin
70	2	Gastric cancer	Camrelizumab	Paclitaxel + Carboplatin
71	2	Esophageal cancer	Pembrolizumab	Paclitaxel + Carboplatin
72	2	Esophageal cancer	Tislelizumab	Paclitaxel
73	2	Gastric cancer	Durvalumab	Paclitaxel + Carboplatin
74	2	Non small cell lung cancer	Tislelizumab	Pemetrexed + Carboplatin
75	2	Non small cell lung cancer	Tislelizumab	Paclitaxel + Carboplatin
76	2	Liver cancer	Camrelizumab	Oxaliplatin + Fluorouracil
77	2	Gastric cancer	Nivolumab	Paclitaxel + Carboplatin
78	2	Non small cell lung cancer	Tislelizumab	Paclitaxel
79	2	Liver cancer	Pembrolizumab	Oxaliplatin + Capecitabine
80	2	Cervical cancer	Toripalimab	Paclitaxel + Cisplatin
81	2	Head and neck cancer	Tislelizumab	Paclitaxel + Carboplatin
82	2	Head and neck cancer	Pembrolizumab	Paclitaxel + Cisplatin
